# Acute COVID-19 and LongCOVID syndrome – molecular implications for therapeutic strategies - review

**DOI:** 10.3389/fimmu.2025.1582783

**Published:** 2025-04-17

**Authors:** Krzysztof Piotr Michalak, Amelia Zofia Michalak, Alicja Brenk-Krakowska

**Affiliations:** ^1^ Laboratory of Vision Science and Optometry, Physics and Astronomy Faculty, Adam Mickiewicz University in Poznań, Poznań, Poland; ^2^ Faculty of Medicine, Poznań University of Medical Sciences, Poznań, Poland

**Keywords:** SARS-CoV-2, autophagy, inflammation, NOX, Nrf2, calcium signaling, nitric oxide, HIF-1α

## Abstract

Severe Acute Respiratory Syndrome Coronavirus 2 (SARS-CoV-2) has been recognized not only for its acute effects but also for its ability to cause LongCOVID Syndrome (LCS), a condition characterized by persistent symptoms affecting multiple organ systems. This review examines the molecular and immunological mechanisms underlying LCS, with a particular focus on autophagy inhibition, chronic inflammation, oxidative, nitrosative and calcium stress, viral persistence and autoimmunology. Potential pathophysiological mechanisms involved in LCS include (1) autoimmune activation, (2) latent viral persistence, where SARS-CoV-2 continues to influence host metabolism, (3) reactivation of latent pathogens such as Epstein-Barr virus (EBV) or cytomegalovirus (CMV), exacerbating immune and metabolic dysregulation, and (4) possible persistent metabolic and inflammatory dysregulation, where the body fails to restore post-infection homeostasis. The manipulation of cellular pathways by SARS-CoV-2 proteins is a critical aspect of the virus’ ability to evade immune clearance and establish long-term dysfunction. Viral proteins such as NSP13, ORF3a and ORF8 have been shown to disrupt autophagy, thereby impairing viral clearance and promoting immune evasion. In addition, mitochondrial dysfunction, dysregulated calcium signaling, oxidative stress, chronic HIF-1α activation and Nrf2 inhibition create a self-sustaining inflammatory feedback loop that contributes to tissue damage and persistent symptoms. Therefore understanding the molecular basis of LCS is critical for the development of effective therapeutic strategies. Targeting autophagy and Nrf2 activation, glycolysis inhibition, and restoration calcium homeostasis may provide novel strategies to mitigate the long-term consequences of SARS-CoV-2 infection. Future research should focus on personalized therapeutic interventions based on the dominant molecular perturbations in individual patients.

## 1 Introduction

Since its emergence in late 2019, Severe Acute Respiratory Syndrome Coronavirus 2 (SARS-CoV-2) has posed an unprecedented challenge to global health. While the acute phase of Coronavirus Disease 2019 (COVID-19) is increasingly well understood, growing attention has turned toward long-term complications. LongCOVID Syndrome (LCS) affects millions of patients worldwide and manifests as a constellation of multisystem symptoms persisting months after viral clearance, with mechanisms that remain elusive (1, 2).

Recent studies have highlighted the ability of SARS-CoV-2 proteins to interfere with key intracellular pathways, including autophagy, mitochondrial dynamics, oxidative and nitrosative stress responses, calcium signaling, and the regulation of transcription factors such as HIF-1α and Nrf2 (3–11). These perturbations are believed to contribute to both viral persistence and immune dysregulation. However, no study to date has thoroughly examined how viral proteins simultaneously affect multiple cellular pathways, each controlled by its own feedback mechanisms, and how the disruption of these systems may interact to worsen or prolong disease. This lack of a systems-level perspective limits our understanding of how SARS-CoV-2 causes long-term cellular dysfunction.

This review aims to fill this gap by providing an integrative analysis of how viral proteins reprogram intracellular signaling, with particular focus on autophagy, Nrf2-mediated antioxidant defenses, calcium signaling, and mitochondrial function. We further examine how dysregulation of feedback mechanisms between these pathways may lead to sustained cellular stress and impaired resolution of inflammation. A better understanding of these interdependencies could inform novel therapeutic strategies for both acute and post-acute COVID-19.

## 2 LongCOVID syndrome

LongCOVID-19 Syndrome (LCS) is characterized by a variety of health complications affecting multiple organ systems, including the respiratory, cardiovascular, hematologic, genitourinary, gastrointestinal, and neurologic systems (1, 2). The underlying cause of these symptoms is thought to be the persistent activation of the immune system (1, 12). Various definitions have been proposed for LCS. Some authors propose to distinguish between Post Acute COVID-19 (4-12 weeks after infection), Post COVID-19 (more than 12 weeks) and Long COVID-19 which includes both of these terms (2). Another term used is Post-Acute Sequelae of SARS-CoV-2 Infection (PASC). However, it should be noted that this classification is somewhat artificial and has a negligible impact on the current analysis.

Previous analyses of the LCS problem have focused on describing the immunologic, molecular, and morphologic changes in the organs or tissues that are damaged or dysregulated (1, 2). The criteria for the diagnosis of LCS have also been discussed in the context of different symptoms and affected organs (1, 2). The features of hyperinflammation, coagulation disorders, oxidative stress and tissue-dependent damage are presented as major endpoint features of LCS. However, little attention has been paid to the initial pathobiochemical and molecular changes in cellular metabolism that lead to the observed end-effects. Assuming a large variety of different LCS types, the question arises as to what is the reason for the prolonged duration of symptoms in patients after SARS-CoV-2 infection and to what extent the molecular and immunological changes observed during and after COVD-19 are the reason for the symptoms of LCS.

Four main alternatives can be considered as the reason for LCS:

1. Induction of a type of autoimmunity [29,30]. The autoantibodies, after binding to some receptors on the surface of the cells, induce its metabolic reprogramming causing regulatory dysfunctions.
2. COVID-19 infection induces other chronic pathogens that were already present in the host cells (e.g. Epstein-Barr virus, cytomegalovirus) (13, 14) to go to the “higher level” of their body infiltration causing the magnification of their earlier latent symptoms. The development of intracellular infections can be attributed to intracellular metabolic or immunological changes that facilitate pathogen propagation. Autophagy inhibition induced by SARS-CoV-2 proteins (15–18) appears to be a possible facilitator of disease progression.
3. The SARS-CoV-2 virus remains in the cells after recovery, it is not completely eliminated and enters the chronic active state (19, 20). The patient’s symptoms are to a high degree the direct metabolic consequences of the presence of the virus in the host cells.
4. Neither SARS-CoV-2 nor other pathogens or autoimmunity are present in the host cell as an activating stimulus. The presence of SARS-CoV-2, other intracellular pathogens and autoimmunity have been excluded as initiating elements of LCS. The metabolic and immunological alterations have been shown to be a consequence of the pathological state (e.g. chronic mitochondrial dysfunction, chronic microcirculatory disturbances), and the organism is unable to leave this state and return to a healthy states characterized by proper activity of all metabolic pathways and transcription factors. The only possible explanation for LCS is the inability of host cells to exit the deregulated state.

It is important to note that the first three proposed causes of LCS may overlap in individual patients, and the fourth cause can only be considered after the first three have been ruled out.

Autoimmune disorders and the induction of other pathogens will be discussed in a basic outline, as it is dedicated to a separate review. However, a detailed analysis will focus on the impact of individual viral proteins on metabolism, assuming that LongCovid-19 syndrome is generated by an active chronic viral infection and interference of viral proteins with host metabolism, as is the case with other viruses that enter a state of chronic active infection, such as EBV (21–26), CMV (27), HCV (28, 29), HSV (30, 31). In this case, the key to Long Covid syndrome therapy is to understand the interaction of virus proteins with the host’s metabolism in order to reverse the disorders and restore the immune system’s ability to remove virus-laden cells or restore the ability of host cells to clear the virus.

### 2.1 COVID-19 activates multiple autoimmune diseases

Autoimmunity appears to be a important component of the symptoms associated with LCS, as evidenced by the presence of various autoantibodies in a significant proportion of patients with both LCS and COVD-19 [29,30]. Numerous reports have indicated the presence of various autoantibodies following infection with SARS-CoV-2 (32). These autoantibodies associated with the SARS-CoV-2 infection include autoantibodies against type I interferons, lupus anticoagulant (LAC), antinuclear antibodies (ANA), rheumatoid factor (RF), p- and c-antineutrophil cytoplasmic antibodies (pANCA, cANCA), anticardiolipin antibodies (ACL), anti-Ro52 antibodies and anti-phosphatidylserine antibodies (32). Antiphospholipid antibodies were mainly represented by IgG ACL (48%), followed by IgM ACL (21%), especially in COVID-19 positive patients (32). As reported by Zhou et al. (33), autoantibodies were found to be directed against 12 different host antigens. These immune molecular elements included antinuclear antibodies (ANA), anti-SSA/Ro antibodies, anti-Scl-70 antibodies, and anti-U1-RNP antibodies.

Vojdani et al. (34) demonstrated by ELISA that certain monoclonal antibodies directed against the spike protein of SARS-CoV-2 showed reactivity with several self-antigens, including glutamate decarboxylase-65 (GAD-65), mitochondrial proteins, phospholipids, and hepatocyte microsomes.

As reported by Chang et al. (35), multiple IgG autoantibodies were found in hospitalized patients with confirmed cases of COVID-19. Some of these autoantibodies have been shown to contribute to the formation of proinflammatory immune complexes, primarily on endothelial cell surfaces. This has the potential to lead to vascular inflammation and thrombosis. For example, increased formation of neutrophil extracellular traps (NETs) was found in patients with COVID-19 who also had vasculitis (35). This phenomenon was found to be associated with significant neutrophil activation and production of proinflammatory NETs, which were observed to contain nucleic acids, histones, and various inflammatory peptides or proteins (35). Autoantibodies identified in this study included anti-C1q antibodies, previously observed in systemic lupus erythematosus, as well as anti-β2GP1, anti-bactericidal/permeability-increasing protein (BPI), and anti-ACE-2 antibodies. In addition, 60-80% of all hospitalized patients with confirmed COVID-19 expressed at least one anti-centromere antibody (ACA), a finding also reported in other autoimmune diseases, most commonly in CREST syndrome (35).

As also found by Wang et al. (36), IgG isotypes from patients with anti-GM-CSF, anti-CXCL-1 or anti-CXCL-7 autoantibodies have the potential to block the signaling of these proteins. In addition, increased antibody-dependent cellular phagocytosis was observed in Raji B cells or Jurkat T cells due to the presence of anti-CD38 or anti-CD3ε autoantibodies (36). Furthermore, the study established a correlation between autoantigens such as NXPH-1, PCSK-1, SLC2A10 and DCD and markers of COVID-19 severity, including D-dimer, ferritin, C-reactive protein and lactate, which were observed to increase in cases of severe COVID-19 (36). In addition, there have been reports of an association between viral-induced autoimmunity and COVID-19 based on the mechanism of molecular mimicry. Certain proteins on the surface of SARS-CoV-2 have been shown to be biochemically similar to those found in host cells (37, 38). Therefore, it is plausible that some patients with COVD-19 may manifest cross-reactive immunologic responses analogous to those observed in other pathologic conditions, such as acute post-streptococcal glomerulonephritis or rheumatic endocarditis.

The research on the existence of autoimmunity in LCS patients is less numerous, but significant. According to Ampudia et al. (39), there is persistent autoimmune activation and a proinflammatory state in LCS. For example, the frequency of β2-glycoprotein-1 (β2-GP1) IgM autoantibodies, classically described in antiphospholipid syndrome, was higher in LCS patients compared to pre-pandemic controls (39). The same study also found that 19 of 33 acute cases of COVID-19 and 21 of 33 LCS patients expressed at least one autoantibody. According to Ampudia et al., LCS patients have higher levels of circulating naive B cells, which are a known source of autoantibodies. Furthermore, patients with LCS had very high levels of circulating proinflammatory cytokines such as IFN-α, TNF-α, G-CSF, IL17A, IL-6, IL1-β, and IL-13, but also a decrease in interferon-γ-induced protein-10 (IP-10) (39). Persistent dysregulation of IL-6 (one of the major proinflammatory cytokines in COVID-19) was found to be associated with generalized fatigue, sleep disturbance, depression, and anxiety and is one of the major proinflammatory molecules associated with the development of autoinflammatory responses and autoimmunity via pre-existing B lymphocytes (39). The clinical presentation of LCS has been found to be influenced by other molecular elements, including IL-1β, TNF-α, IFN-γ, IL-10, IL-2, C-reactive protein, MCP-1, serum amyloid-A and metabolites of the kynurenine pathway (39).

In conclusion, the autoimmunity that develops during and after SARS-CoV-2 infection is a significant problem that needs to be analyzed independently for different types of autoantibodies. Further research is needed to determine which antibodies are “silent” (i.e., do not react with any proteins or receptors in host cells) and which are active, i.e., react with some proteins and thereby induce or inhibit specific molecular pathways. The issue of autoimmunity in LCS requires dedicated research and analysis, with a focus on the inhibition of persistent induced inflammation. Treatment should aim to gradually reduce the levels of inflammatory antibodies, thereby facilitating recovery.

### 2.2 Activation of other active chronic infections

A recent reports describe that SARS-CoV-2-induced infection may lead to reactivation of EBV (40–42). Other viruses that belong to the Herpesvirus family: HHV-6 and HHV-7 were also shown to be reactivated in patients infected with SARS-CoV-2 (42). The effect of reactivation of pathogens is in line with studies indicating that the SARS-CoV-2 virus disrupts various immune functions, including the blocking of autophagy in numerous molecular mechanisms (16). Another mechanism observed for the SARS-CoV-2 NSP15 protein is the inhibition of type 1 interferon production (43). It has been observed that a virus with a mutated NSP15 induced normal interferon production, while the wild type induced a broad immune response including the induction of ER stress and upregulation of over 2,800 genes, including networks associated with activating the unfolded protein response and the proinflammatory response associated with viral pathogenesis (43). It indicates the important role of this viral protein in weakening antiviral immunity.

It is important to acknowledge that the chronic active viremia is not exclusive to the SARS-CoV-2. Various other viruses, including Epstein-Barr virus (EBV) (21–26), cytomegalovirus (27), Ebola virus (44), Zika virus (45), enteroviruses (46), Coxsackie (47–49) and measles virus (50), have also been documented to enter the active chronic state and induce various health complications. EBV, a common virus in the general population, often progresses to active chronic infection. Studies employing blood PCR in healthy individuals by various authors suggest a population burden of 18-30% (51–54) and up to 80% in the older adult population in Qatar (55). Population analyses for CMV are similar. A study by Huifen Li et al. (56) demonstrates that active chronic CMV infection can persist for decades in older individuals and leads to a threefold increase in blood levels of the pro-inflammatory IL-6. The study further suggests that the virus occupies immune cells, including monocytes, CD4 and CD8 lymphocytes. In a group of subjects aged 70-79 years, 46% had CMV DNA detected in monocytes. As in the case with SARS-CoV-2, both viruses have been postulated to induce autoimmunity and hypercoagulation (57–64) including disseminated intravascular coagulation (DIC) (65–67).

EBV, CMV and Coxsackie B3 have also been reported to inhibit autophagy (16, 68, 69), suggesting a potential for these two viruses and SARS-CoV-2 to facilitate each other’s spread within the body. Other viruses that chronically block autophagy may also contribute to this process. Naendrup et al. (14) reported that in a cohort of 117 critically ill COVID-19 patients, EBV reactivation was detected in 16% and CMV in 9%. Reactivations were more frequent in patients receiving corticosteroids (58% for EBV, 55% for CMV) (14). While targeted treatment with ganciclovir improved survival in CMV patients (83% vs. 0% without treatment), rituximab did not show a significant effect on EBV outcomes. These findings suggest that while viral reactivations are common in severe cases of COVID-19, further study is necessary to determine their clinical impact and the benefits of targeted treatment.

### 2.3 Sars-Cov-2 still persisted in the cells despite negative tests

The mounting body of evidence indicates the presence of SARS-CoV-2 RNA and protein in a broad spectrum of tissue types, collected weeks or months after the onset of acute SARS-CoV-2 infection. The viral RNA or protein has been identified in the majority of organs and tissues, including the liver, stomach, tonsils, gallbladder, and lungs (20, 70–74). The preponderance of evidence for the existence of a SARS-CoV-2 reservoir in individuals with LCS stems from three sources: tissue biopsy studies, studies of SARS-CoV-2 proteins in plasma and studies using features of the adaptive immune response to infer the presence of a SARS-CoV-2 reservoir in tissues.

One of the earliest reports concerning the prolonged positive PCR tests at recovery patients was published by Lan et al. (19). Four members of the hospital staff were prescribed antiviral treatment (75 mg of oseltamivir taken orally every 12 hours) due to a positive diagnosis of SARS-CoV-2 infection. Subsequently, two consecutive negative RT-PCR test results were obtained. The time from the onset of symptoms to recovery ranged from 12 to 32 days. Thereafter, the subjects were instructed to adhere to a five-day home quarantine protocol. Subsequent RT-PCR tests, conducted between 5 and 18 days later using two distinct PCR kits, yielded positive results, despite the absence of symptoms as determined by clinical examination and chest CT findings.

Cheung et al. (20) detected nucleocapsid protein (NP) of SARS-CoV-2 in various organs including the liver, colon, lymph nodes, appendix, ileum, haemorrhoids and gallbladder, in five cancer patients who had recovered from the disease. These patients had tested negative for SARS-CoV-2 between 9 to 180 days prior. Notably, viral antigen was detected in all tissues of two patients, suggesting widespread multi-organ involvement from the viral infection. It is noteworthy that in the colon, viral antigen was detected exclusively in normal colonic crypts and polyps, while it was absent from neoplastic tissues.

In separate study Tao et al. (75) demonstrated that gastrointestinal (GI) symptoms were exhibited by a considerable proportion of patients with COVID-19. Furthermore, the study revealed that seven out of fifteen patients with confirmed cases of SARS-CoV-2 RNA positivity in their stool samples exhibited negative results in their respiratory samples and did not manifest any gastrointestinal symptoms. *In vitro* transcriptional analysis employing a SARS-CoV-2-infected cell model revealed that the 3’ end of the viral genome was significantly more covered compared to the 5’ end, suggesting an indication of active viral replication and infection (75). Furthermore, the presence of opportunistic pathogens, including Collinsella aerofaciens, Collinsella anakaei, Streptococcus infantis, and Morganella morganii, was observed in higher concentrations in SARS-CoV-2-positive fecal samples (75). Additionally, these samples exhibited an augmented capacity for nucleotide biosynthesis and amino acid and carbohydrate metabolism (glycolysis). However, the level of short-chain fatty acid-producing bacteria was diminished (75). These findings suggest a potential significant role for GI dysbiosis in SARS-CoV-2 patients; however, the direction of causality remains uncertain, as the study does not elucidate whether the dysbiosis is a consequence or a cause of the infection.

In a separate study, Tejerina et al. (76) utilized RT-PCR to detect SARS-CoV-2 in plasma, urine, and stool samples from patients with LCS. The patients were examined 39 to 67 days after their initial diagnosis of COVD-19. The study’s participants included 29 patients who had reported symptoms such as fatigue, myalgia, dyspnea, inappropriate tachycardia, and low-grade fever for more than 4 weeks after their initial diagnosis of COVD-19. The disease manifested as mild in 55% of cases, while 13 patients (45%) exhibited positive plasma RT-PCR results and 51% were positive in at least one RT-PCR sample (plasma, urine, or stool). Notably, 18 patients (62%) were undergoing antiviral treatment using lopinavir/ritonavir and hydroxychloroquine, which led to a substantial improvement in their health status (76). The findings indicate a pattern of persistent or recurrent/intermittent SARS-CoV-2 viremia in approximately 50% of patients with non-specific symptoms, which could be interpreted as LCS.

Further research on viral persistence in the human body can be found in (77). The analyses presented herein demonstrate that the probability of long-term viral persistence in the human body subsequent to a diagnosis of COVD-19 infection is relatively high, irrespective of the severity of the disease. Consequently, this possibility should be addressed with caution during the analysis of patients exhibiting LCS symptoms. A comprehensive review of the literature reveals that persistent viral infection may be responsible for approximately 50-70% of LCS patients.

## 3 Molecular basis for the LongCOVID syndrome caused by SARS-CoV-2 persistence

**Figure 1:**
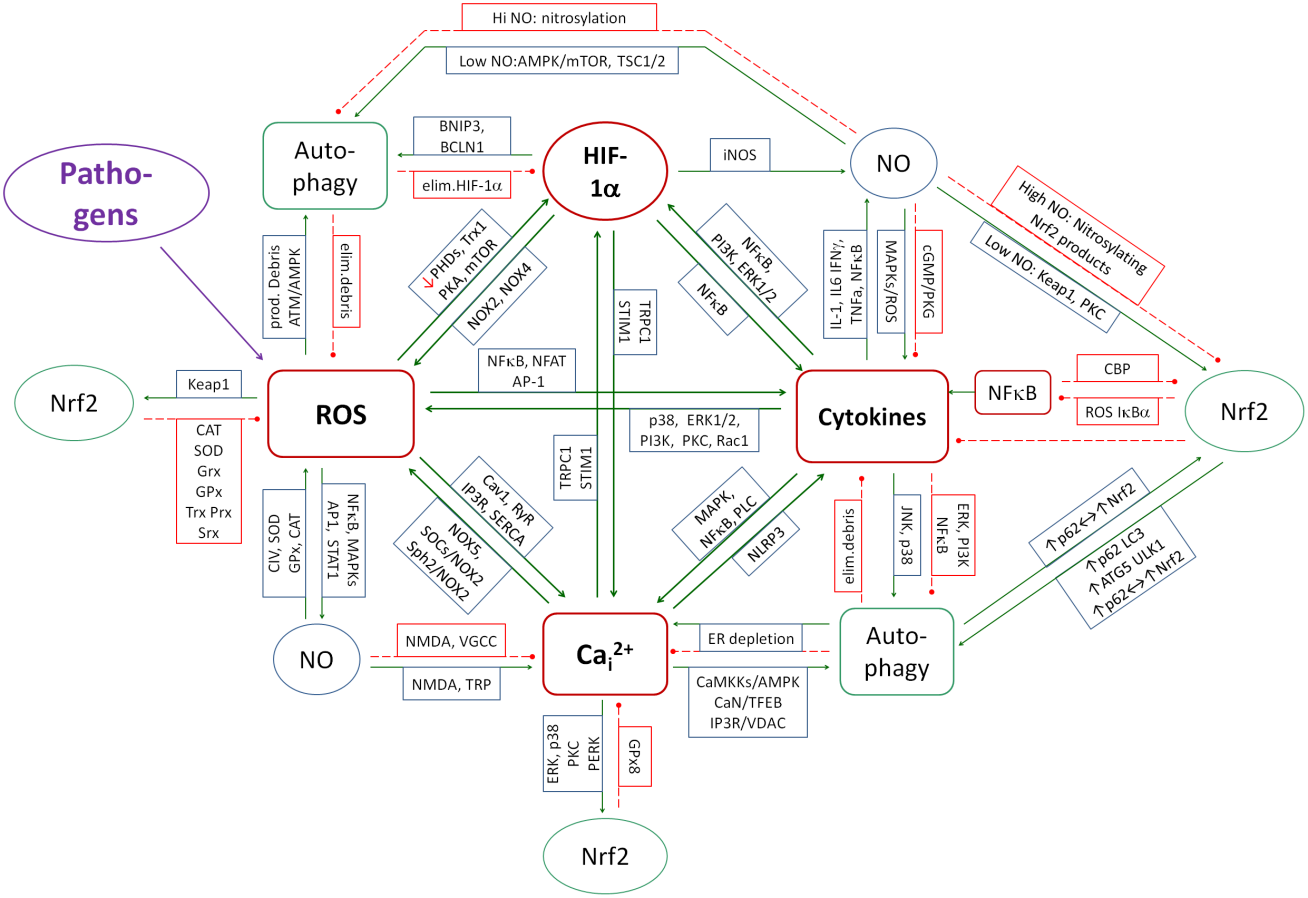
A diagram of the couplings that contribute to development of inflammation. The system of mainly positive couplings between inflammation (cytokines), ROS, Ca_i_^2+^ and HIF-1α forms the core of inflammation induction. NO is partly positively and partly negatively coupled to the core elements and contributes at low concentrations to the control of inflammation and at high concentrations to its amplification. Nrf2 and autophagy create the system of mainly negative couplings that reduce and control inflammation, ROS production, Ca_i_^2+^ and HIF-1α. Green solid arrows - activation, red dashed lines - inhibition.

To understand the mechanisms underlying the metabolic effects of SARS-CoV-2 on host cell metabolism, it is imperative to elucidate the mechanisms of inflammation regulation, which consist of feedbacks between cytokines, oxidative stress, nitrosative stress, calcium stress, reticuloendoplasmic (ER) stress, HIF-1α, autophagy, and Nrf2. These feedbacks have been described by Michalak et al. (78). The disruption of these feedback loops by SARS-CoV-2 is a critical factor in understanding the mechanisms by which it induces LCS syndrome. A particular focus is placed on autophagy, as its disruption has been identified as a key factor in the development of both acute hyperinflammatory and chronic active COVID-19 (16, 18, 79–84) as well as many other diseases such as e.g. autoimmune and neurodegenerative diseases (85–89). A diagram of the couplings in inflammation control between the mentioned elements based mainly on the review by Michalak et al. (78), is shown in **Figure 1**. The analysis of the interaction of individual viral proteins on the metabolism in the context of the above-mentioned feedbacks allows us to understand the multifaceted mechanisms of the development of LongCovid-19 syndrome, as well as the reason for the therapeutic difficulties of this condition. This multiple coupling system will therefore be briefly discussed.

### 3.1 Couplings between cytokines, NOX, NO, Ca_i_^2+^ and HIF-1α

From the point of view of control theory, it is not easy to design a system that, when exposed to a variety of pathogens, excites itself to levels high enough to fight the pathogen, but not so high as to exceed the threshold of self-destruction. Evolution has programmed a system in which cytokines, NOXs, HIF-1α and Ca_i_^2+^ form a mutual positive feedback system. When induced by a pathogen, they reinforce each other to fight the pathogen. On the other hand, autophagy and Nrf2 are regulators of this spiral, turning on mechanisms that inhibit this coupling spiral. HIF-1α and NO have a dual effect, as their action reinforces the inflammatory spiral in some conditions and inhibits it in others. When analyzing the effect of SARS-CoV-2 proteins on metabolism, one must keep this regulatory system in mind, since the activities of all these elements are disrupted by the viral proteins. The total effect of the virus on metabolism must take into account not only the sum of the individual interactions but also the mutual feedbacks between these elements, which amplifies the total effect.

**Figure 2:**
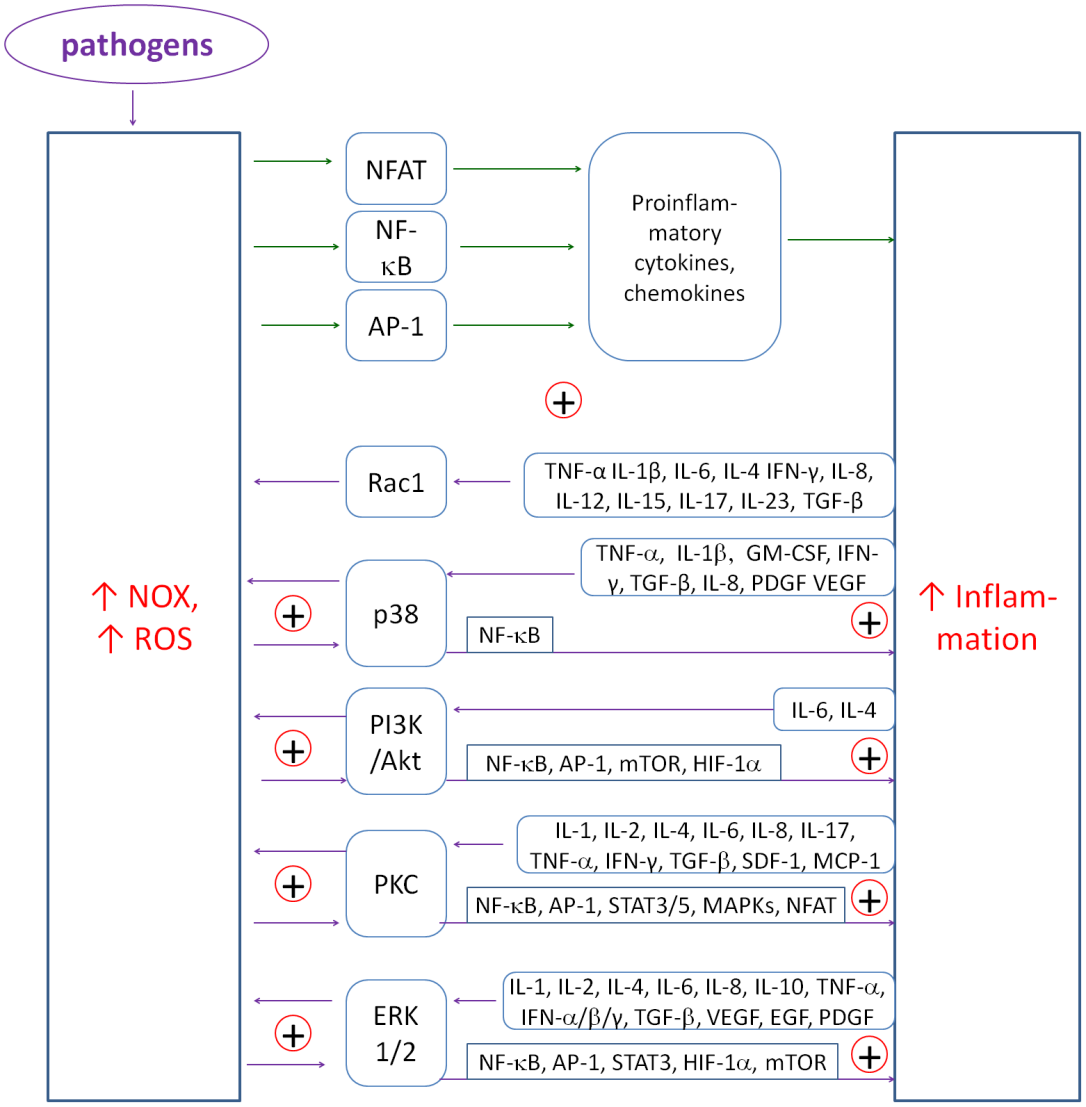
The diagram of positive couplings between NOX-derived ROS and inflammatory cytokines. The multiplicity of positive couplings forms the flywheel of inflammatory induction. ROS induce NF-κB, NFAT and AP-1 to produce mainly proinflammatory cytokines and chemokines. Conversely, cytokines induce NOXs to produce ROS mainly through p38, PI3K, PKC and ERK1/2 kinase pathways.

The primary coupling is between cytokines and NADPH oxidases, which produce H_2_O_2_. These couplings form the main flywheel of inflammation in the fight against pathogens (see **Figure 2**). NOX-derived ROS stimulate the transcription of pro-inflammatory genes via factors such as NF-κB (90–92) or NFAT (93). Conversely, priming of NOXs occurs in response to a variety of cytokines such as TNF-α (94–96), IL-1β (97), IL-6 (98), IL-4 (99), IFN-γ (100), IL-8 (101), IL-12 (102), IL-15 (103), IL-17 (104), IL-23 (105), TGF-β (106–110). ROS also amplify inflammatory signals by activating kinase pathways such as p38 MAPK or ERK1/2, creating a complex network of interdependencies. Chronic maintenance of such couplings can lead to uncontrolled chronic inflammation and potential tissue damage. **Figure 2** shows a simplified diagram of these couplings and the mediating role of transcription factors and kinase pathways.

Ca_i_^2+^ and HIF-1α are additional elements that drive the inflammatory spiral (see **Figure 1**). ROS-induced increases in Ca_i_^2+^ levels activate the NLRP3 inflammasome, leading to IL-1β production and enhanced inflammation (111). In turn, inflammatory cytokines increase Ca²^+^ influx into cells via MAPK, PLC (phospholipase C), and NF-κB, establishing a positive feedback loop. PLC produces IP3 and DAG. IP3 binds to IP3 receptors in the endoplasmic reticulum, resulting in the release of Ca^2+^ ions from the ER into the cytoplasm (112), whereas DAG activates transient receptor potential canonical (TRPC) channels, resulting in the influx of Ca^2+^ ions from the extracellular space (113).

HIF-1α activity depends on multiple feedbacks with ROS, NO, cytokines and Ca^2+^. It has a dual function: it is protective for mitochondria under hypoxic conditions and pro-inflammatory in response to stressors. The protective effect is mainly due to inhibition of PDH (pyruvate dehydrogenase) and pyruvate influx into mitochondria, thereby reducing Krebs cycle turnover and mito-ROS production (114). The positive feedback between HIF-1α and succinate/fumarate acts as an amplifier of this relationship (115) as succinate and fumarate contribute to the stabilization of HIF-1α, while activation of HIF-1α leads to an increase in their concentration in mitochondria by blocking PDH, activating glycolysis (GLL), gluconeogenesis (GNG), and the glucose transporter GLUT-1. ROS stabilize HIF-1α by inhibiting its degradation and increasing its stability (116, 117). Stabilization of HIF-1α, in turn, induces NOX expression (118), which further increases ROS production. In addition, ROS activate NF-κB, which increases HIF-1α transcription (119). In turn, HIF-1α activates inflammation mainly through NF-κB (120). Proinflammatory cytokines such as TNF-α and IL-1β induce HIF-1α through activation of the PI3K/Akt and NF-κB pathways (121–124). In addition, HIF-1α modulates the expression of calcium channels such as TRPC1 and STIM1, which increase Ca^2+^ influx into the cytoplasm (125–128). The increase in Ca^2+^ can activate calmodulin-dependent proteins that increase HIF-1α activity, closing the positive feedback loop (129). In summary, HIF-1α exerts a protective effect against mito-stress. However, under conditions of chronic inflammation, its interactions with ROS, Ca_i_^2+^ and cytokines promote the perpetuation of inflammation.

### 3.2 The role of kinase pathways

**Figure 3:**
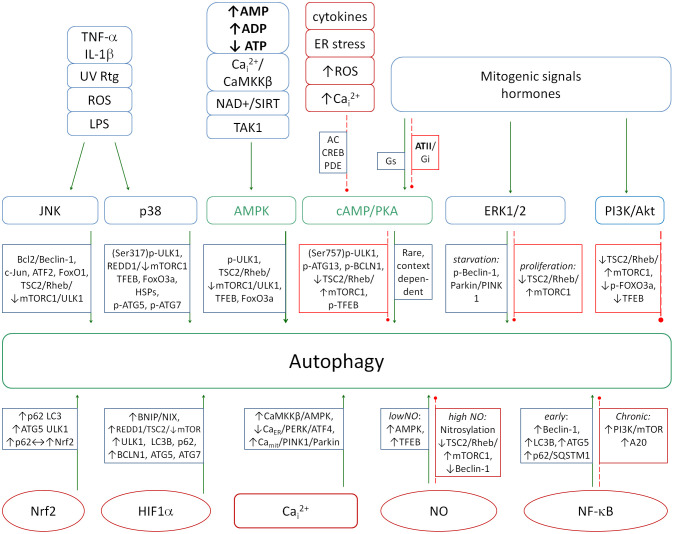
A diagram showing the action of major signaling pathways (top row) and selected molecules (bottom row) on the induction of autophagy. The proper balance between the pro- and anti-autophagic pathways is necessary to properly combat pathogens. Increased autophagy inhibition leads to the chronicity of infections and is a hallmark of several autoimmune diseases. Green solid arrows - activation, red dashed lines - inhibition.

Kinase pathways, including MAPK (p38, ERK1/2, JNK), PI3K/Akt, JAK/STAT, AMPK and cAMP/PKA, have been identified as central regulators of inflammation and autophagy processes. The p38, ERK1/2, JNK and PI3K/Akt pathways are mainly proinflammatory, while AMPK and cAMP/PKA are anti-iflammatory. The JAK/STAT pathway is actually a series of pathways depending on the type of STAT protein. Some STATs are pro-inflammatory and some are anti-inflammatory. A diagram of the action of individual signaling pathways and selected metabolites on autophagy is shown in **Figure 3**. This diagram shows the complexity of the autophagy regulation. Even a small imbalance of this control can disrupt the process leading to cellular pathology. It is worth noting that in the case of a severe inflammation, NO changes its effect from pro-autophagic to anti-autophagic, which can have an important impact on the final balance of this process.

The p38 MAPK pathway is known to be activated by various environmental stressors, including oxidative stress, hypoxia, UV or ionizing radiation, and osmotic disturbances (130), Additionally, the pathway is stimulated by inflammatory factors such as TNF-α (131, 132), IL-1β (133, 134) and TGF-β (135, 136)) as well as by pathogens including bacterial lipopolysaccharides (LPS) (137), which activate toll-like receptors (TLR). In response, p38 induces the expression of inflammatory genes (138–142) and promotes autophagy (143) as a protective mechanism.

ERK1/2 controls cell proliferation and the production of inflammatory cytokines (144–147). It is activated in response to inflammatory stimuli such as IL-17A, IL-1β (134, 148), bacterial LPS (149), or growth factors (e.g., VEGF, EGF) (150) Excessive activation of this pathway can lead to pathological inflammatory conditions, including psoriasis, rheumatoid arthritis, and Crohn’s disease (151, 152). ERK1/2 can induce autophagy by activating the Beclin-1 protein, although this effect is believed to occur at least partially as a consequence of PI3K/Akt pathway inhibition (153, 154). Conversely, under conditions that favor cell growth and proliferation, ERK1/2 may suppress autophagy by promoting mTOR activity via the TSC2/Rheb/mTORC1 axis, a mechanism that is particularly prominent in cancer (155) and is also implicated in neurodegenerative disorders (156).

The JNK pathway, by activating the transcription factor AP-1, increases the expression of pro-inflammatory mediators such as IL-6, IL-8, and TNF-α as well as chemokines that attract immune cells to the site of inflammation (157–159). In macrophages and neutrophils, JNK promotes the production of ROS, which contribute to the destruction of pathogens (160). The JNK pathway also plays a role in the resolution of inflammation by promoting apoptosis in dysfunctional cells, thereby limiting excessive inflammation (161), and by promoting autophagy which reduces the debris-mediated inflammation (161, 162). Its chronic activation has also been implicated in autoimmune diseases (163).

PI3K/Akt is a pathway that modulates pro- and anti-inflammatory processes, with the capacity to promote the production of pro-inflammatory cytokines (IL-6, IL-1β) (164–169) while concomitantly reducing excessive inflammation by inducing anti-inflammatory cytokines such as IL-10 (170–172) Inhibition of FOXO by Akt leads to the inhibition of autophagy, production of antioxidant enzyme and apoptosis, thereby promoting a state of chronic inflammation (173, 174). The hyperactivation of this pathway has been implicated in the development of various autoimmune diseases such as rheumatoid arthritis, inflammatory bowel disease and asthma (175, 176).

In contrast to the previously mentioned pathways, AMPK and cAMP/PKA function as a significant anti-inflammatory regulators (177–181) by inhibiting the activation of NF-κB (a potent pro-inflammatory transcription factor) (182, 183). PKA is also a potent inflammation resolving factor being reduced in acute phase of inflammation by increased PDE4 activity (breaking cAMP to AMP) (184) and being active when the inflammation finishes thus activating other resolving molecules (184, 185) and activating phagosomes (186). Additionally, both pathways enhance mitochondrial function and reduce levels of reactive oxygen species (ROS) levels (187–191) and reduce oxidative stress (187, 192).

AMPK promotes autophagy (193, 194) but influence of PKA depends on the metabolic content (195). A deficiency in AMPK has been associated with chronic inflammatory conditions including obesity and type 2 diabetes (196, 197) and deficiency of PKA is associated with multiple diseases including neurodegeneration, hypertension, type 2 diabetes, depression and anxiety (183, 198–201).

The equilibrium between the activities of the aforementioned pathways is pivotal in the modulation of inflammation. From the perspective of autophagy, certain pathways are stimulatory, while others are inhibitory, depending on the metabolic context. Consequently, an imbalance in the ratio of these pathways, resulting in excessive inhibition of autophagy, may serve as a significant catalyst for the transition of the regulatory system toward a state of chronic or hyperinflammation.

### 3.3 Concentration dependent roles of NO

Nitric oxide (NO) produced by iNOS (inducible nitric oxide synthase) exerts both pro- and anti-inflammatory effects, depending on its concentration and metabolic context. Pro-inflammatory cytokines such as IL-1, IL-6, IFN-γ, TNF-α and pro-inflammatory transcription factors: NF-κB (202), AP-1 (203), NFAT (204), STAT1 (205), and HIF-1α (206) induce the expression of iNOS and NO production (202). Subsequently, NO can then interact with O_2_^-^, to form toxic peroxynitrite (ONOO^-^). NO is known to have anti-inflammatory properties, mainly through the mechanisms of cyclic GMP (cGMP) (207–209) and S-nitrosylation of proteins (210). This effect is also partly related to the inhibition of NF-κB by cGMP (211). Another anti-inflammatory mechanism involves the induction of apoptosis in activated macrophages leading to a reduction in the inflammatory response (212, 213). Conversely, NO has been shown to activate inflammation by activating MAPK kinases (ERK, JNK, p38) through the induction of oxidative stress (e.g., by peroxynitrite) (214). In addition, excess NO has been demonstrated to exacerbate oxidative stress through nitrosylation and subsequent inhibition of antioxidant enzymes including superoxide dismutase (SOD), glutathione peroxidase (GPx) and catalase (CAT) (215). Furthermore, NO can also nitrosylate specific proteins within the endoplasmic reticulum, resulting in the accumulation of misfolded proteins, ER stress and subsequent NOX activation (216). The final NO regulatory effect appears to be concentration dependent and should be analyzed with caution in this context.

### 3.4 The regulatory role of autophagy and Nrf2

The main mechanisms that control inflammatory process and prevent self-destruction are autophagy and Nrf2. Autophagy removes abnormal proteins that induce inflammation through various mechanisms. These abnormal proteins include both viral proteins and abnormally folded cellular proteins resulting from ER stress. It is important to note that autophagy is a complex process involving more than 100 different proteins, and the block or inhibition of even one of these proteins by a pathogen protein can contribute to the loss of cellular regulatory capacity for autophagy. An imbalance in autophagy regulation can lead to its inhibition contributing to chronic and severe inflammation (217–219). This observation is significant when considered in the context of the fact that many intracellular pathogens chronically inhibit autophagy, thereby preventing their complete clearance from the cell (220–225).

Regulation of autophagy is closely linked to cytokines, ROS, NO, HIF-1α and Ca_i_^2+^. Cytokines and ROS activate autophagy via pathways such as JNK (226), p38 (139, 227–230) and AMPK (231–233). However, in the context of inflammation, there is a delicate balance between the activatory and inhibitory influences of diverse signaling pathways on the autophagy process. The ERK1/2 pathway (234, 235) and NF-κB represent the primary inhibitory elements (236, 237). NF-κB functions in a positive feedback loop with the proautophagic p38 pathway (228–230, 238–240), thereby establishing a balance in intensity of autophagy.

Intracellular Ca^2+^ works as autophagy activator. Elevated calcium levels activate AMPK and inhibit mTOR, thereby promoting autophagy (241). In addition, Ca^2+^-activated calcineurin activates lysosomal biogenesis by phosphorylating TFEB (transcription factor EB).

HIF-1α also modulates autophagy in response to hypoxia by activating autophagy genes. In general, HIF-1α activates the autophagy (242, 243) and autophagy reduces the HIF-1α activity by degrading this molecule (244). NO exerts a dual regulatory effect on autophagy, functioning as both an activator and an inhibitor depending on its concentration and the specific cellular context. At physiological levels, NO promotes autophagy maintaining homeostasis. Moderate levels of NO stimulate autophagy mainly through the AMPK-mTOR pathway (245). In addition, NO increases ROS production by activating pathways such as JNK, which further enhances autophagic responses under conditions of oxidative stress. However, in pathological conditions such as chronic inflammation or excessive oxidative stress, excessive NO has been shown to inhibit autophagy via S-nitrosylation of key autophagic proteins such as ATG4, which impairs autophagosomal membrane elongation (246). NO has also been observed to nitrosylate JNK1 (blocking its autophagy-activating function) (247) and IKKβ, reducing AMPK phosphorylation while activating mTORC1, leading to autophagy suppression (248, 249). In summary, excess NO can significantly contribute to the maintenance of chronic inflammation and the persistence of chronic pathogens within the cell by both inhibiting autophagy and reducing Nrf2-induced antioxidant response. Chronic inflammation further exacerbates autophagy inhibition through sustained activation of NF-κB, which suppresses AMPK and Nrf2 and activates mTOR.

Nrf2 is a key transcription factor responsible for protecting cells from oxidative stress and regulating inflammatory processes. Its activity relies on complex feedbacks with NF-κB, ROS, NO, Ca²⁺ and HIF-1α, making it central to the antioxidant and anti-inflammatory system. Nrf2 is regulated by the protein Keap1, which under healthy conditions binds Nrf2 and targets it for degradation by proteasomes. Under conditions of oxidative stress, reactive oxygen species (ROS) and nitrogen (NO) modify cysteine residues in Keap1, leading to the release of Nrf2 and its translocation to the nucleus (250–253). Nrf2 then activates genes responsible for the production of antioxidant enzymes such as heme oxygenase 1 (HO-1), glutathione S-transferase (GST) and NAD(P)H:quinone oxidoreductase 1 (NQO1) which reduce ROS levels, thereby restoring redox balance. However, at excessive levels of NO and ONOO^-^, Nrf2-induced enzymes (e.g., HO-1, Mn-SOD, catalase, peroxiredoxin II E, glutathione peroxidase-thioredoxin reductase) are damaged or deactivated by nitrosylation, leading to a breakdown of the antioxidant barrier and promoting severe or chronic inflammation (250, 254–258).

Nrf2 also has an inhibitory effect on the cytokine system by blocking the activation of NF-κB and both ROS reduction and HO-1 production mediate this effect (259, 260). Conversely, NF-κB inhibits Nrf2 (261, 262), creating a specific double-negative regulatory system that enhances either the oxidative-inflammatory or antioxidant state, depending on which regulatory scale prevails. In chronic inflammation, decreased Nrf2 and increased NF-κB activity are often observed, leading to a loss of balance between pro- and anti-inflammatory mechanisms.

Intracellular calcium can activate various signaling pathways such as ERK1/2 and p38, which can increase Nrf2 activity by phosphorylating Nrf2 and increasing its stability (263, 264). Nrf2 also affects the regulation of Ca²⁺ in cells, reducing ER stress by increasing the expression of glutathione peroxidase GPx8 in the ER (265), reducing Ca_i_^2+^ by modulating calcium channels (e.g. TRPC, RyR) (266) and increasing the activity of the calcium pump SERCA (266). In summary, Nrf2 and Ca_i_^2+^ work in the classical negative coupling, where Ca_i_^2+^ induces Nrf2 and Nrf2 reduces the calcium and ER stress.

In conclusion, Nrf2 is a key regulator of redox balance and inflammation whose function depends on dynamic interactions with ROS, NO, HIF-1α and Ca²⁺. Disruption of Nrf2 activation leads to an escalation of oxidative stress and inflammation. Interventions aimed at stabilizing Nrf2 are therefore an important therapeutic strategy in inflammatory and degenerative diseases.

## 4 Metabolic alterations generated by SARS-CoV-2

**Figure 4:**
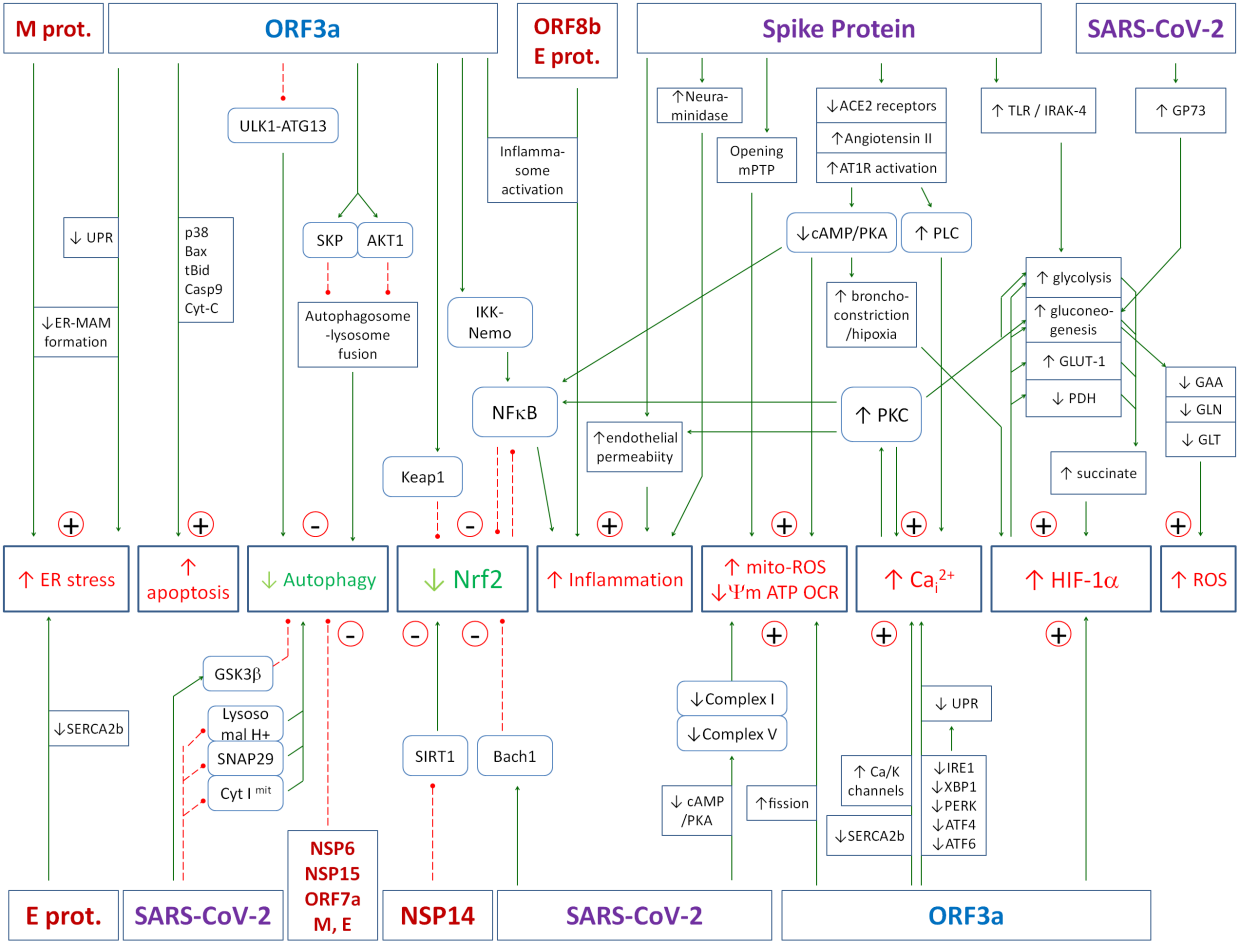
The figure summarizes the effects of the SARS-CoV-2 virus or its individual proteins on key elements of the cell’s antiviral defense. The numerous molecular mechanisms that contribute to the high pathogenicity of the virus, the generation of a hyperinflammatory state, and the propensity to progress to a state of active chronic infection are presented. Details of the various interactions are described in the text of the article. Green solid arrows - activation, red dashed lines - inhibition.

The SARS-CoV-2 genome encodes 29 proteins: 16 non-structural proteins (NSP1-16), 4 structural proteins: envelope protein [E], membrane protein [M], spike protein [S] and nucleocapsid protein [N], and 9 accessory proteins: ORF3a, ORF3b, ORF6, ORF7a, ORF7b, ORF8, ORF9b, ORF9c, and ORF10. A number of mechanisms have been described by which the virus as a whole or its individual proteins reprogram the cell’s metabolism to make it easier for itself to replicate and harder for the host to fight off. The mechanisms largely involve interference with and disruption of the feedback loops described above, leading to hyperinflammation. Key effects include mitochondrial dysfunction, inflammatory activation, inhibition of Nrf2, induction of calcium stress, inhibition of autophagy and induction of HIF-1α. A summary of all viral protein interactions with host cell metabolism discussed in the following subsections is shown in **Figure 4**. Assuming that a significant proportion of LCS patients are those with active chronic viral presence in cells, the following analysis which primarily considers the acute infection state may also be useful in determining treatment strategies for LCS patients.

### 4.1 Upregulation of the angiotensin 1 receptor

The key feature of the S1 protein is its ability to bind to the ACE2 (angiotensin converting enzyme) protein on the surface of cells, allowing it to enter the interior. ACE2 cleaves angiotensin II (ATII) into angiotensin (1-7), a hormone with opposite effects to angiotensin II (267, 268). During severe COVID-19 infection, ACE2 is blocked by the virus and the amount of this protein on the cell surface is reduced, leading to an increase in ATII and a decrease in its antagonist, angiotensin (1-7) (268). Excess ATII is an important factor in initiating a cascade of regulatory abnormalities in the cell (268–271). It acts through the angiotensin receptors AT1, AT2, AT3 and AT4, which are upregulated in COVID-19 (267).

AT receptors belong to the group of GPCRs (G-protein coupled receptors). Two main signaling pathways are involved in GPCR signaling: cAMP and phosphatidylinositol signaling (272). Depending on the type of cell, different types of GPCR receptors can be found on the surface of the cell, that trigger a specific response inside the cell. Most GPCR receptors are capable of activating more than one type of G protein, especially when the receptor is over-activated. This can happen, for example, when the concentration of ATII in the blood increases excessively. Activation of the AT1 receptor causes, among other things, activation of phospholipase C (PLC) and inhibition of adenylyl cyclase (AC) and cAMP/PKA pathway (273), which contribute to hyperinflammation in the course of COVID-19 (274). Activation of PLC leads to an increase in cytosolic Ca^2+^ concentration. Another effect of AT1 receptor overstimulation is the activation of NOX5, NF-κB, IL-6 and STAT3, which exacerbates oxidative stress and causes overproduction of pro-inflammatory cytokines (including IL-6, IL-1β and TNF-α) (268, 275–277).

Downregulation of PKA has several important effects on cellular metabolism. It causes deregulation of mitochondria by increasing electron leakage from cytochromes I and IV (188, 189). It also destabilizes the structure and decreases ATP production in cytochrome V (ATP synthase) (190, 191) and removes the regulatory inhibitory effect of high ATP levels on cytochrome IV (189), which is the natural negative feedback that decreases further ATP production and slows electron flow without its leakage. All of these effects contribute to the decrease in the mitochondrial membrane potential. It is also questionable whether the increased concentration of succinate, as an effect of HIF-1α induction, generates in such conditions the reverse flow of electrons from cytochrome II to I, generating the strong electron leakage in cytochrome I, as described in (188).

Next, the cAMP/PKA pathway causes relaxation of bronchial smooth muscle cells (278), thus, inhibition of AC by excess ATII promotes the contractile state of bronchial smooth muscle. Another effect of the downregulation of the cAMP/PKA pathway is the enhancement of inflammation and the increase of oxidative stress. The anti-inflammatory mechanism is that the CREB-CBP complex formed by CREB phosphorylation by PKA can result in the dissociation of the NF-κB-CBP complex, which blocks the action of NF-κB (183). PKA also inhibits the activation of the pro-inflammatory ERK, AKT, STAT3 and NF-κB pathways through phosphorylation and inhibition of the TNFR1 receptor (185), so down-regulation of PKA activity increases the strength of the coupling between inflammation and oxidative stress. As cAMP/PKA pathway is the important factor initiating the resolution of inflammation (185, 279), the slow recovery process after the disease may also depend on its downregulation. It should be noted that the inhibition of this pathway occurs in the early stage of the disease, so, it may be the important factor initiating and then sustaining the inflammatory-oxidative cascade.

### 4.2 Upregulation of calcium stress by SARS-CoV-2

There are several ways in which SARS-CoV-2 can increase cytosolic calcium. As mentioned above, the first is stimulation of the AT1 receptor by angiotensin II which further activates phospholipase C and its activation leads to an increase in cytosolic Ca^2+^ concentration. Increased Ca_i_^2+^ activates the calmodulin pathway, which has a number of downstream effects, one of which is the activation of the proinflammatory transcription factor NFAT (274). The other important effect of Ca_i_^2+^ is the activation of protein kinase C (PKC). Activation of PKC can lead to many metabolic changes that are involved in the severity of COVID-19. PKC contributes to the activation of NF-κB (280), which subsequently activates HIF-1α, inhibits Nrf2, and inhibits autophagy. PKC may also contribute to the activation of glycogenolysis and gluconeogenesis in the liver (281), which, in conjunction with HIF-1α overactivation, leads to increased glycolysis and increased succinate/fumarate concentration. Another role of PKC is to mediate the stimulation of proinflammatory cyclooxygenase-2 expression by viral spike protein through both calcium-dependent (PKCα) and calcium-independent mechanisms (PKCϵ, NF-κB, ERK, PI3K) (282).

### 4.3 Creating potassium/calcium channels by ORF3a

One of the important capabilities of the viral ORF3a protein is the ability to create ion channels that decrease the concentration of K_i_^+^ and increase Ca_i_^2+^ in the cell (3). This is particularly dangerous in the context of reduced energy production, as more energy amount is required to maintain the ion gradients. Decreased K_i_^+^ concentration leads to decreased membrane potential, which can activate voltage-dependent calcium channels (VDCCc) (283, 284), which further increases Ca_i_^2+^, and one of the possible effects is the activation of calcium-activated potassium channels (KCa), which further decreases K_i_^+^ concentration (285). In the normal state, this process attempts to increase the membrane potential and reduce calcium influx, but in the pathological state, it drives cellular pathology. The above mechanism is postulated to induce apoptosis and necrosis (286, 287).

### 4.4 Reticuloendoplasmic stress caused by ORF3a and M proteins

The other pathway of calcium homeostasis disruption presented by Lee et al. (4) is the ability of SARS-CoV-2 ORF3a and M proteins to disrupt the ER membrane and mitochondrial-associated membrane (MAM) formation. This study demonstrated that ORF3a and M proteins affect the proteomic landscape of the ER. It was also shown that ORF3a-APEX2 constructs used for electron microscopy imaging significantly increased the formation of cubic membranes (CM), also known as convoluted membrane structures. Notably, CM structures were also observed in coronavirus-infected cells (288, 289). In the case of M protein, the ER was clearly disrupted in M-expressing cells and appeared to curl into whorl patterns (also referred to as aggresomes). Their results suggested that the ORF3a and M proteins of SARS-CoV-2 may be major contributors to the formation of ER-derived neoorganelles.

The interactome analysis for these two viral proteins also showed that a large proportion of plasma membrane and endoplasmic reticulum membrane proteins were present in both the ORF3a and M interactomes. This suggests that the function of the ER and especially the MAM is significantly disrupted by these proteins. The disruption of the ER and induction of the unfolded protein response (UPR) by SARS-CoV-2 has been described by several authors (5, 6). The detailed mechanisms include depletion of Ca_ER_^2+^ concentration and increase in Ca_i_^2+^ (290, 291) and one of the mechanisms generating it is a direct interaction between E-protein and SERCA2b pump resulting in a decrease in SERCA-mediated ER Ca^2+^ reloading (7). It has also been postulated that ORF3a is capable of generating calcium channels that could reduce the Ca^2+^ gradient across the ER membrane (292).

### 4.5 Loss of endothelial monolayer integrity caused by Spike protein

Loss of the pulmonary endothelial barrier is one of the most dangerous effects caused by SARS-CoV-2 and several pathways are involved in this effect, most of which are related to S1 protein activity. The first pathway is increased PKC activity which can increase endothelial permeability (8). Even low levels of PKC activation can reverse cell chirality through PI3K/AKT signaling and alter the organization of junctional proteins between cells, leading to significant changes in endothelial permeability that may promote increased inflammation and pulmonary edema (8). The second pathway is the inhibition of adenylyl cyclase (AC) by ATII, one of the effects of which is bronchoconstriction contributing to hypoxia (278). In the experiment by Jana et al. (9), incubation of S1 protein with human pulmonary endothelial arterial cells resulted in the disruption of endothelial barrier function, an increase in the levels of numerous inflammatory molecules (VCAM-1, ICAM-1, IL-1β, CCL5, CXCL10), an increase in mitochondrial reactive oxygen species (ROS) and a slight increase in glycolytic reserve capacity. The effect of S1 was enhanced by the hypoxia that occurs during severe COVID-19 due to pulmonary hyperinflammation and reduced blood oxygen saturation, as hypoxia increases the ability of S1 to reduce ACE2 receptor levels.

S1 protein-mediated loss of endothelial monolayer integrity was also observed experimentally by Buzhdygan et al. using an advanced 3D microfluidic human blood-brain barrier model (10). They also found similar pro-inflammatory cytokine responses induced by spike protein, which are thought to contribute to the loss of membrane integrity (10). ICAM-1 and VCAM-1, key players in immune cell transendothelial migration following inflammatory challenge, were observed to be upregulated by S1 and associated with reduced barrier integrity (9).

### 4.6 Neuraminidase activity of the spike protein

One of the likely S1 activities is the enzymatic activity of neuraminidase (NEU) (293). Zhang et al. (293) used advanced 3D techniques to describe the similarity of S1-protein to the neuraminidase from influenza A and B viruses. Neuraminidase is the enzyme that cleaves sialic acid on the surface of leukocytes, which is the key stimulus for neutrophil activation to activate inflammation, cytokine storm, NOX-induced ROS production and neutrophil extracellular traps (NETs) formation (293).

In severe systemic inflammatory responses such as sepsis and COVID-19, neutrophils are central to organ damage. Hyperactivated neutrophils infiltrate vital organs and release cytotoxic molecules such as proteases, cytokines, ROS and NETs (294, 295). Although inflammatory mediators are essential for fighting infection, they can also damage the host cells (296).

Neuraminidase is one of the major activators of neutrophils, so neuraminidase inhibitors can be treated as important factors in inhibiting the inflammatory cascade. Formiga et al. (297) demonstrated the role of neuraminidase-1 (NEU1) and matrix metalloproteinase-9 (MMP-9) in triggering the hyperinflammatory state in COVID-19 patients. Lipopolysaccharide (LPS) has been shown to induce host membrane‐associated NEU activation in murine or human macrophages and dendritic cells (298). Upon LPS binding to toll-like receptor 4 (TLR4), matrix metalloproteinase 9 (MMP-9) induces NEU activity and contributes to the NF-κB-induced response of macrophages, suggesting a role for these enzymes in cell activation (298, 299). In another paper, Formiga et al. (300) showed that *in vitro* treatment of whole blood with the viral neuraminidase inhibitors oseltamivir or zanamivir, inhibited the activity of human neuraminidases as well as the exacerbated neutrophil response. These drugs also reduced neutrophil activation and increased survival in mice.

The neuraminidase inhibitor (NI) clinical trial presented by Wu et al. (301) showed that NI treatment reduced the mortality rate (5.7% vs. 10.3%) and the critical illness conversion rate (14.1% vs. 19.7%) compared with the non-NI group. They also showed that levels of N-acetylneuraminic acid and neuraminidase (predominantly the NEU3 isoform) were elevated in COVID-19 subjects and recovered 1 month after infection, suggesting increased desialylation in COVID-19 patients.

### 4.7 SARS-CoV-2 and mitochondria

Mitochondrial dysfunction, subsequent pathogenesis and multi-organ failure have been associated with COVID-19 infection (302). Functional mitochondrial analysis of COVID-19 peripheral blood mononuclear cells (PBMCs) revealed mitochondrial dysfunction, increased glycolysis and high mitokine levels (303). The presence of the oxidative stress in mitochondria in SARS-CoV-2 infected cells was confirmed by increased carbonyl content in the mitochondrial fractions in SARS-CoV-2-infected lung tissue lysates (304), suggesting that SARS-CoV-2 infection induces an oxidative stress environment in the lung cells in general, which also affects mitochondrial proteins. A reduction in mitochondrial respiration was also observed, as indicated by a loss of oxygen consumption rate (OCR) in isolated mitochondria from SARS-CoV-2-infected hamster lungs. Proteomic analysis also revealed specific deficits in the mitochondrial ATP synthase (Atp5a1) within complex V and in the ATP/ADP translocase (Slc25a4) (304). The other determinant of mitochondrial oxidative stress was the increased staining for 4-hydroxynonenal (4-HNE), a major end-product of lipid peroxidation (304).

SARS-CoV-2 infection also causes a reduction in the mitochondrial electron flow at several cytochrome complexes indicating a general virus-induced impairment of mitochondrial function. A lower basal OCR in the presence of pyruvate and malate was observed, indicating impaired complex I activity in the SARS-CoV-2-infected patients (304). In a complex V assay, a significant decrease in the ATP synthesis was observed in the SARS-CoV-2-infected lung mitochondria suggesting a possible impairment of the complex V ATP synthesis function (304).

The other important parameter of mitochondrial energy production is the mitochondrial membrane potential ΔΨm. The discussion of the energy production in mitochondria and the relationships between ΔΨm, Ca_i_^2+^ and K_i_^+^ concentrations, opening of mitochondrial permeability transition pores (mPTPs), and oxidative stress has been presented by Michalak et al. (305). In short, intracellular calcium and ROS cause the opening of mPTPs, which causes the efflux of protons from the intermembrane space and the decrease of ΔΨm. This leads to decreased energy production which may contribute to decreased Ca_i_^2+^ removal from the cytoplasm and increased Ca_i_^2+^, as this process is highly energy consuming.

Impaired complex I activity in the SARS-CoV-2 infected patients (304) may play a protective role to some extent, as it reduces the hydrogen entry into the ETC. However, electron leakage can vary depending on the detailed metabolic context. PKA inhibition caused by angiotensin II increases in electron leakage (188). On the other hand, metformin, a weak complex I inhibitor, reduces electron leakage in COVID-19 patients (306–311). However, other metabolic properties of metformin may also be involved in this protection (306, 307).

### 4.8 Disrupting the mitochondria by ORFs

ORF3a has an important disruptive effect on the mitochondrial function. ORF3a-induced ROS production and cell death appears to be a highly conserved activity (3, 312, 313), as these effects are observed in both fission yeast and human A549 and 293 T cells (3). However, the detailed molecular mechanisms of ORF3a-induced ROS production are not fully understood. ORF3a-mediated apoptosis has been described to be both mitochondria-dependent (287) and independent (286, 314). Exemples of mito-dependent proapoptotic effects include the activation of caspase-8, the increase in caspase-9 levels and release of cytochrome c from the mitochondrial membrane. Increased Bax oligomerization, Bid to tBid conversion, higher p53 levels and increased p38 activity mediate these effects (315–318).

Another study showed that ORF3a promotes mitochondrial fission, which can lead to the destabilization of normal physiological processes within the mitochondria. Increased fission is associated with impaired mitochondrial function and altered energy production (11). It has also been shown that ORF3a, ORF9b, ORF9c and ORF10 induce significant mitochondrial and metabolic reprogramming in A549 lung epithelial cells. While all four ORFs caused mitochondrial fragmentation and altered mitochondrial function, only ORF3a and ORF9c induced a marked structural change in mitochondrial cristae (11). ORF9b, ORF9c and ORF10 induced largely overlapping transcriptomes. In contrast, ORF3a induced a distinct transcriptome, including the downregulation of numerous genes for proteins with critical mitochondrial functions and morphology (11). Notably, reduced amino acid metabolism and increased metabolism of some lipids was induced by ORF3a (11).

### 4.9 Inhibition of Nrf2 by SARS-CoV-2

The Nrf2-induced cellular antioxidant response is an important element in the overall balance of the cellular antiviral response. In viral infections, oxidative cellular damage may be associated with inhibition of the Nrf2 pathway (319). The expression of Nrf2-related antioxidant genes has been shown to be suppressed in biopsies from COVID-19 patients (320). Rodrigues et al. (321) showed reduced nuclear accumulation of Nrf2 and expression of Nrf2 targeted genes in endothelial cells exposed to COVID-19 serum. In addition, these cells showed increased expression of Bach-1, a negative regulator of Nrf2 that competes with Nrf2 for DNA binding. All events were prevented by tocilizumab, the IL-6 receptor blocker, suggesting that IL-6 is the major cytokine involved in the inhibition of Nrf2-induced antioxidant defense (321). However, more complex regulatory mechanisms may have led to this effect as presented in **Figure 1**. Further research has shown that the inhibition of Nrf2 is due, at least in part, to the activity of the viral protein ORF3a. ORF3a promotes degradation of Nrf2 by recruiting Keap1, thereby attenuating cellular resistance to oxidative stress and facilitating ferroptotic cell death (322). Downregulation of Nrf2 and its dependent genes by SARS-CoV-2 infection exacerbates pulmonary inflammation and disease and therefore activation of Nrf2 appears to be an important element of the therapeutic approach during both chronic and acute SARS-CoV-2 infection (323, 324). In another study Shilei et al. showed that the SARS-CoV-2 NSP14 protein interacts with the catalytic domain of the NAD-dependent deacetylase sirtuin 1 (SIRT1) and inhibits its ability to activate the Nrf2/HO-1 pathway (325).

In conclusion, Nrf2 is the major down-regulator of most of the positive couplings that cause hyperinflammation and the inhibition of Nrf2 by viral proteins may be an important factor that amplifies the excessive oxidative stress and inflammatory couplings that cause the severe course of the disease or sustain the chronic state. In particular, reduced Nrf2 activity contributes to increased NF-κB activity and thus to the inhibition of autophagy. A reduction in Nrf2 activity is therefore an important pathway leading to an excessive positive feedback between cytokines, NOX, Ca_i_^2+^ and HIF-1α, resulting in their excessive levels, leading to a cytokine storm and a state of hyperinflammation. In the case of chronic active SARS-CoV-2, it contributes to the chronic state of LCS.

### 4.10 Activation of HIF-1α

HIF-1α plays a important role in the progression of SARS-CoV-2 infection by influencing metabolism, inflammatory responses, and viral replication. The viral protein capable of activating HIF-1α is ORF3a (313). Accumulation of HIF-1α results from both increased expression and inhibited proteasomal degradation (326). The primary cause of HIF-1α activation is hypoxia due to disruption of the alveolar-capillary barrier, leading to pulmonary edema, immune cell infiltration, and impaired gas exchange (9, 327). This damage can be induced by the S1 subunit of the viral spike protein, even in the absence of other viral components (9, 304). In addition, activation of AT1 receptors increases intracellular calcium, leading to bronchoconstriction and further hypoxia. The pathways involved are inhibition of adenylyl cyclase (328) and activation of phospholipase C, which increases Ca_i_^2+^ and then activates PKC.

HIF-1α drives glucose metabolism by promoting glycolysis and gluconeogenesis and these processes are upregulated during SARS-CoV-2 infection (307, 329–337). Glycolysis is enhanced by upregulation of glucose transporters (GLUT-1) and activation of hexokinase, pyruvate kinase, lactate dehydrogenase and aldolase (304, 338–341). Gluconeogenesis is increased by hepatocyte infection, activation of enzymes such as PEPCK, and increased levels of GP73 (335, 336). These processes contribute to hyperglycemia and the accumulation of metabolites such as lactate and succinate, which further enhance HIF-1α activation and inflammation. It is worth noting that the natural inhibitor of glycolysis is citrate, so citrate can be considered as a natural support in the therapy of COVID-19 and other viruses that increase glycolysis.

Stabilization of HIF-1α also promotes viral replication. Proteins such as ORF3a enhance HIF-1α activity, leading to increased viral replication and cytokine production. Excess lactate from glycolysis inhibits mitochondrial antiviral signaling (MAVS), further facilitating viral replication (331). This creates a feedback loop in which HIF-1α enhances the effects of SARS-CoV-2 within the host. Targeting HIF-1α has therapeutic potential, but general inhibition should be avoided because HIF-1α has a the protective effect on mitochondria by reducing mitochondrial electron leakage and mito-stress (329). Instead, therapies should focus on specific effects, such as glucose metabolism and inflammatory responses, driven by HIF-1α activation.

### 4.11 Inhibition of autophagy by ORF3a, NSP6, ORF7a, NSP15, M and E proteins

The final metabolic effect of SARS-CoV-2 discussed here is the inhibition of autophagy (16). Recent studies have shown that different SARS-CoV-2 proteins inhibit autophagy through multiple pathways (16). NSP6 inhibits lysosomal acidification, the formation of acidic autolysosomes, and the formation of the hybrid pre-autophagosomal structure (HyPAS) - a precursor structure in the autophagy pathway that integrates various components from the ER, Golgi apparatus, and endosomes. Next, NSP6 supports autophagosome formation but inhibits autophagosome maturation (79–81). ORF3a inhibits the formation of acidic autolysosomes by sequestration of the HOPS component VPS39. When VPS39 is sequestered by ORF3a, the fusion process of the autophagosome with the lysosome is disrupted. Next, ORF3a inhibits PI3K complex II assembly by sequestrating its component UVRAG resulting in impaired endosome maturation. Finally, ORF3a inhibits lysosomal acidification and promotes lysosomal exocytosis, facilitating viral release (18, 82, 83). ORF7a inhibits lysosomal acidification and the formation of acidic autolysosomes (84). NSP15 inhibits autophagy initiation (84), M protein inhibits autolysosome formation (18, 84) and E protein inhibits autophagosome maturation and autolysosome formation (84).

The other viral proteins that interfere with antiviral defense through autophagy are ORF8 and NSP13. ORF8 promotes autophagic degradation of MHC-I (342), leading to a reduction in the amount of MHC-I on the surface of infected cells. As a result, infected cells are no longer recognized by CD8⁺ T cells, which impairs their elimination. NSP13 promotes autophagic degradation of TBK1 (343). TBK1 is a key activator of IRF3, which is required for the expression of the type I antiviral interferons IFN-α and IFN-β.

Interestingly, ORF3a of SARS-CoV-2, but not SARS-CoV has the ability to block autophagy (18, 344). This is an important piece of information that points to the critical role of autophagy inhibition in exacerbating the severity of SARS-CoV-2 compared to SARS-CoV infection.

### 4.12 Upregulating the inflammation

All of the discussed effects of SRAS-CoV-2 on host cell metabolism generally contribute to increased levels of inflammation. However, direct activating effects have also been observed. Nie et al. (345) investigated SARS-CoV-2 genes and found that ORF3a activates the proinflammatory NF-κB pathway by interacting with IKK-β (beta subunit of the IκB kinase complex) and NEMO (also known as IKK-γ) and by enhancing the interaction of IKK-β-NEMO, thereby positively regulating NF-κB activity. Other viral proteins are also capable of activating inflammation. ORF3a, ORF8b and E proteins have been reported to enhance activation of the inflammasome, leading to increased secretion of IL-1 and IL-18, and subsequent pathological changes associated with inflammation. Similarly, the NSP9 and NSP10 proteins of SARS-CoV-2 induce overproduction of IL-6 and IL-8, which are the major causes of the cytokine storm in COVID-19 patients (346).

### 4.13 Inhibition of Interferon production

Interferon I is one of the main lines of antiviral defense. Activation of IFN-I production is a complex process regulated by multiple signaling pathways that detect the presence of viral RNA or DNA in cells. Interferon regulatory factors 3 and 7 (IRF3, IRF7) are key transcription factors that regulate interferon production. The main signaling pathways are RIG-I-like receptors (RLRs), Toll-like receptors (TLRs) and stimulator of interferon gene (STING). Among others NEMO (347, 348), MAVS (349, 350), and NLRP6 (351) modulate type I IFN production and inflammasome activation (352–355), whereas TRIM18 and TRIM29 suppress macrophage activation and IFN production (354, 356).

SARS-CoV-2 has several proteins which act on the type I IFN pathway. The proteins NSP1 (357, 358), NSP3 (359), NSP5 (357), NSP6 (357), NSP10 (360), NSP12 (359), NSP15 (43, 357, 358), NSP16 (358, 361), ORF3b (362, 363), ORF6 (357), ORF7b (357), ORF9b (364), ORF10 (357, 365) M (366) and N proteins (367), act on the type I IFN pathway either by inhibiting transcription or by acting on effector mechanisms. The inhibition of interferon production and the delayed host response to the presence of the virus in cells leads to significant molecular perturbations, as in the case of NSP15, which causes the upregulation of over 2800 genes including networks associated with the activation the unfolded protein response (UPR) and the proinflammatory response associated with viral pathogenesis (43).

The above studies suggest a completely different role for viral proteins during the incubation period of the disease, when the immune response is inhibited, as opposed to the late phase of the disease, when there is hyperactivation of the immune system and a cytokine storm, as described in the earlier sections of this article. It also suggests the need for different therapeutic targets during the period of potential disease incubation and during the period of its late, severe course. With regard to LongCOVID-19, it is necessary to study to what extent in this phase it is necessary to stimulate the production of interferons and activate inflammatory processes, and to what extent it is necessary to inhibit them. It is not excluded that the optimal therapeutic strategy might be the simultaneous activation of interferon I production pathways, e.g. by blocking TRIM18 or TRIM29 (354, 356), to improve the detection of virus-infected cells and to inhibit the inflammatory response, e.g. by inhibiting NF-κB, which may help to restore proper Nrf2 and autophagy activity.

## 5 Summary

Analysis of the interaction of different viral proteins on cellular metabolism generally indicates a synergistic effect in increasing inflammation, oxidative stress, nitrosative stress, mitochondrial stress and calcium stress in the cell which explains the tendency of the virus to generate the hyperinflammatory state. Reciprocal positive feedbacks between these elements enhance the pro-inflammatory effect of the virus. In addition, inhibition of autophagy and Nrf2 disrupts two important regulatory mechanisms that prevent excessive inflammation and all of the above stresses. The number of couplings disrupted by viral proteins is so large that eradication of the infection is sometimes beyond the capabilities of the regulatory system. Understanding, how these couplings work, is essential for planning therapeutic strategies not only for COVID-19 but also for many other infections, both acute and chronic.

Analysis of the feedback loops shows that the regulatory system is extremely complex. The multitude of positive feedback loops suggests that treatment strategies with a single drug acting on a single therapeutic pathway or transcription factor have little chance of bringing the patient out of hyperinflammatory or chronic state, as the remaining active positive feedback loops continue to drive inflammation and oxidative stress, leading all the time, but only slightly weaker, to the destruction of the system. Analyzing this system, it can be expected that only the alleviation of at least some of the positive feedbacks can bring about a rapid improvement in the patient’s health. According to the author, a special attention should be paid to the activation of autophagy and Nrf2. If autophagy is blocked, the cell has no alternative way to get rid of viruses and misfolded proteins. Its activation seems to be essential for therapeutic success. Activation of Nrf2 also seems to be an important element as it can lead to inhibition of NF-κB and reduction of inflammation, which is particularly important in a state of hyperinflammation. Another underestimated therapeutic approach, according to the author, is the inhibition of glycolysis, e.g. with citric acid, a natural glycolysis inhibitor (368). This could be an important natural element to protect mitochondria from electron leakage, mito-stress, succinate accumulation and thus reduce HIF-1α activation. Research is also needed into the use of drugs or herbs that reduce calcium and reticuloendoplasmic stress, as these are significantly involved in the development of hyperinflammation or LCS. In particular, activation of the cAMP/PKA pathway, which is blocked by ATII, is worth considering as a therapeutic target, as it is likely to be an important element generating mitochondrial stress.

A more complex case is LCS caused by the development of autoimmune processes as a result of COVID-19 infection. The profile of metabolic abnormalities induced by different autoantibodies can vary considerably from patient to patient. In addition, conditions in which SARS-CoV-2 has induced chronic active infection with other pathogens (e.g. EBV) may require the use of appropriate diagnostic tests and individualized therapies depending on the type of pathogen. For example, EBV and cytomegalovirus are capable of causing coagulopathies, including disseminated intravascular coagulation (DIC) (65–67). However, it appears that the profile of metabolic disturbances in such cases will be similar to that of COVID-19, as the process of pathogen persistence is very often associated with the blockade of autophagy by these pathogens (220–225), which further leads to the induction of oxidative, nitrosative, Ca_i_^2+^ and ER stress. The concomitant induction of NF-κB leads to the inhibition of Nrf2.

An important conclusion derived from the analysis of interdependent feedback mechanisms is that influencing a single component of the system inevitably triggers changes throughout the entire network. This highlights the need to differentiate between the direct effects of a particular influence and the indirect consequences that propagate through other interconnected elements.

Looking ahead, future research should aim to mathematically characterize each connection within the network graph by formulating appropriate differential equations or functional relationships. A representative dynamic model utilizing ordinary differential equations, which captures the response of Nrf2, Keap1, Srxn1, and GSH under oxidative stress, was proposed by Hiemstra et al. (369). Once each interaction is quantitatively described, it will become feasible to construct a comprehensive system of equations capable of simulating the behavior of the network, both under normal conditions and in response to disruptive agents such as viral proteins or pharmacological interventions. This, in turn, would provide a foundation for optimizing therapeutic strategies through mathematical modeling.

To achieve this level of system complexity, future collaboration with experts in computational modeling and systems biology will be essential. Their expertise in analyzing the self-regulatory dynamics of multidimensional systems will be critical in developing accurate models of metabolic regulation.

## Glossary

A20: Tumor necrosis factor alpha-induced protein 3
ACE2: Angiotensin-converting enzyme 2
AKT1: RAC-alpha serine/threonine-protein kinase
AMPK: AMP-activated protein kinase
AP-1: Activator protein 1
AT1 receptor: Angiotensin II type 1 receptor
ATII: Angiotensin II
ATF2: Activating Transcription Factor 2
Bach1: BTB and CNC Homology 1
Bcl2: B-cell lymphoma 2
Beclin-1: Bcl-2-interacting coiled-coil protein
Bid: BH3-interacting domain death agonist
BNIP: Bcl-2/adenovirus E1B 19-kDa-interacting protein
CAT: Catalase
CaMKKβ: Calcium/calmodulin-dependent protein kinase kinase beta
cAMP: Cyclic adenosine monophosphate
CCL5: C-C motif chemokine ligand 5 (RANTES)
cGMP: Cyclic guanosine monophosphate
CMV: Cytomegalovirus
CREB: cAMP Response Element-Binding Protein
CXCL10: C-X-C motif chemokine ligand 10
DAG: Diacylglycerol
DIC: Disseminated Intravascular Coagulation
EBV: Epstein-Barr Virus
EGF: Epidermal growth factor
ER: Endoplasmic reticulum
MAM: mitochondrial-associated membrane
ERK1/2: Extracellular signal-regulated kinase 1/2
ETC: Electron transport chain
FoxO1: Forkhead box O1
GAA: glucogenic amino acids
GLN: glutamine
GLT: glutamate
GP73: Golgi protein 73
GPCR: G protein-coupled receptor
GLUT-1: Glucose transporter 1
GPx: Glutathione peroxidase
GSH: Reduced glutathione
GSK3β: Glycogen Synthase Kinase 3 Beta
HIF-1α: Hypoxia-inducible factor 1-alpha
HMOX1: Heme oxygenase 1
HO-1: Heme oxygenase-1
HOPS: Homotypic fusion and protein sorting complex
HSP: heat shock protein
ICAM-1: Intercellular adhesion molecule 1
IFN: Interferon
IL: Interleukin
IRAK-4: Interleukin-1 Receptor-Associated Kinase 4
IRF3: Interferon Regulatory Factor 3
IKKβ: IκB kinase beta
IP3: Inositol 1,4,5-trisphosphate
IRE1: Inositol-Requiring Enzyme 1
IRF3: interferon regulatory factor 3
JAK/STAT: Janus kinase/signal transducer and activator of transcription pathway
JNK: c-Jun N-terminal kinase
Keap1: Kelch-like ECH-associated protein 1
LC3: icrotubule-associated protein 1 light chain 3
LCS: Long Covid-19 Syndrome
LPS: Lipopolysaccharide
MAM: mitochondrial-associated membrane
MAPK: Mitogen-activated protein kinase
MHC-I: Major histocompatibility complex class I
MMP-9: Matrix metalloproteinase-9
mPTP: Mitochondrial permeability transition pore
mTOR: Mechanistic target of rapamycin
NEMO: NF-κB essential modulator
NET: Neutrophil extracellular trap
NEU: Neuraminidase
NFAT: Nuclear Factor of Activated T-cells
NF-κB: Nuclear factor kappa-light-chain-enhancer of activated B cells
NIX: BNIP3L, BCL2/adenovirus E1B 19kDa-interacting protein 3-like*;* NOX, NADPH oxidase
NO: Nitric oxide
Nrf2: Nuclear factor erythroid 2-related factor 2
OCR: Oxygen consumption rate
ONOO^−^: Peroxynitrite
p38: p38 mitogen-activated protein kinase
p62: Sequestosome 1
Parkin: Parkin RBR E3 ubiquitin-protein ligase
PDGF: Platelet-Derived Growth Factor
PDH: Pyruvate dehydrogenase
PEPCK: Phosphoenolpyruvate Carboxykinase
PERK: Protein kinase RNA-like ER kinase
PHD: Prolyl hydroxylase domain-containing protein
PI3K: Phosphoinositide 3-kinase
PINK1: PTEN-induced kinase 1**;** PKA, Protein kinase A
PKC: Protein kinase C
PLC: Phospholipase C
Rac1: Ras-related C3 botulinum toxin substrate 1
REDD1: Regulated in Development and DNA Damage responses 1 (DDIT4)
Rheb: Ras homolog enriched in brain
ROS: Reactive oxygen species
RyR: Ryanodine receptor
SERCA: Sarco/endoplasmic reticulum Ca^2+^-ATPase
SIRT: NAD-dependent deacetylase Sirtuin
SKP: S-phase kinase-associated protein 1
SNAP29: Synaptosomal-Associated Protein 29
Srxn1: Sulfiredoxin-1
SOD: Superoxide dismutase
STAT: Signal transducer and activator of transcription
TBK1: TANK-binding kinase 1
TFEB: Transcription factor EB
TGF-β: Transforming growth factor-beta
TLR: Toll-like receptor
TNF-α: Tumor necrosis factor-alpha
TRPC: Transient receptor potential canonical channel
TSC2: Tuberous Sclerosis Complex 2
VCAM-1: Vascular cell adhesion molecule-1
VEGF: Vascular endothelial growth factor
VPS39: Vacuolar Protein Sorting 39
ULK1: Unc-51 Like Autophagy Activating Kinase 1
UPR: Unfolded protein response
UVRAG: UV Radiation Resistance-Associated Gene
XBP1: X-box Binding Protein 1
